# Effect of the Complex Allele p.[Ile148Thr;Ile1023_Val1024del] in Cystic Fibrosis and Tracing of a Founder Effect in Mexican Families

**DOI:** 10.3390/life14111445

**Published:** 2024-11-07

**Authors:** Namibia Guadalupe Mendiola-Vidal, Cecilia Contreras-Cubas, Francisco Barajas-Olmos, José Rafael Villafan-Bernal, Ana Lucia Yañez-Felix, Humberto García-Ortiz, Federico Centeno-Cruz, Elvia Mendoza-Caamal, Carmen Alaez-Verson, Juan Luis Jiménez-Ruíz, Tulia Monge-Cázares, Esther Lieberman, Vicente Baca, José Luis Lezana, Angélica Martínez-Hernández, Lorena Orozco

**Affiliations:** 1 Immunogenomics and Metabolic Diseases Laboratory, Instituto Nacional de Medicina Genómica, Secretaría de Salud, Tlalpan, Mexico City 14610, Mexico; namibia.m.v@gmail.com (N.G.M.-V.); ccontreras@inmegen.gob.mx (C.C.-C.); fbarajas@inmegen.gob.mx (F.B.-O.); joravibe@hotmail.com (J.R.V.-B.); draanaluciayanezfelix@gmail.com (A.L.Y.-F.); hgarcia@inmegen.gob.mx (H.G.-O.); fcenteno@inmegen.gob.mx (F.C.-C.); jljimenez@inmegen.gob.mx (J.L.J.-R.); tmonge@inmegen.gob.mx (T.M.-C.); 2 Maestría en Ciencias Médicas, Posgrado de Maestría y Doctorado en Ciencias Médicas, Odontológicas y de la Salud, Sede: Hospital General Dr. Manuel Gea González, UNAM, Coyoacán, Mexico City 04510, Mexico; 3 Investigador por México, Consejo Nacional de Humanidades, Ciencia y Tecnología (CONAHCYT), Benito Juárez, Mexico City 03940, Mexico; 4 Posgrado de Alta Especialidad en Medicina Genómica, Sede: Instituto Nacional de Medicina Genómica, Facultad de Medicina UNAM, Coyoacán, Mexico City 04510, Mexico; 5 Clinical Area, Instituto Nacional de Medicina Genómica, Secretaría de Salud, Tlalpan, Mexico City 14610, Mexico; emendoza@inmegen.gob.mx; 6 Genomic Diagnostic Laboratory, Instituto Nacional de Medicina Genómica, Secretaría de Salud, Tlalpan, Mexico City 14610, Mexico; calaez@inmegen.gob.mx; 7 Human Genetic Department, Instituto Nacional de Pediatría, Secretaría de Salud, Coyoacán, Mexico City 04530, Mexico; estherlieberman@yahoo.com.mx; 8 Rheumatology Department, Hospital de Pediatría, CMN Siglo XXI IMSS, Cuauhtémoc, Mexico City 06720, Mexico; vicbaca@infinitummail.com; 9 Cystic Fibrosis Clinic and Pulmonary Physiology Laboratory, Hospital Infantil de México Federico Gómez, Secretaría de Salud, Cuauhtemoc, Mexico City 06720, Mexico; lezana_doc@yahoo.com.mx; 10 Asociación Mexicana de Fibrosis Quística, A.C. Benito Juarez, Mexico City 03700, Mexico

**Keywords:** complex allele, CFTR gene, founder effect, Mexican families, p.(Ile148Thr), I148T, p.(Ile1023_Val1024del), 3199del6

## Abstract

Cystic fibrosis (CF) is a rare autosomal recessive disease most commonly affecting the Caucasian population. CF diagnosis can be a challenge due to the large spectrum of pathogenic variants in the CFTR gene and the effects of complex alleles. Next-generation sequencing has improved our understanding of the contribution of these complex alleles to the wide spectrum of CF clinical symptoms and to the response to medications. Herein, we studied nine CF patients from six unrelated families carrying the complex allele p.[Ile148Thr;Ile1023_Val1024del] with a frequency of 0.18%. All patients were from Central Mexico. This complex allele was found *in trans* with Class I and II pathogenic variants such as p.(Phe508del), and p.(Phe1078Profs*77)]. A targeted search of a dataset of 2217 exomes from healthy individuals revealed that eight individuals (0.18%) carried the p.(Ile148Thr) variant, but only one (0.022%), who was also born in Central Mexico, was a carrier of the complex allele. These findings show an enrichment of this p.[Ile148Thr;Ile1023_Val1024del] complex allele in Mexican CF patients in this region of Mexico. Finally, protein modeling revealed that this complex allele disrupts the secondary structure of the CFTR protein and might alter the ion flow.

## 1. Introduction

Cystic fibrosis (CF, OMIM #219700) is a rare autosomal recessive disorder. To date, 105,000 people live with CF in the world. Its incidence varies from 1/900 to 1/25,000 in different populations. In Mexico, its incidence is estimated at 1/8500 live births annually [1,2,3]. This disease arises from the presence of two pathogenic variants (PVs) *in trans* in the cystic fibrosis transmembrane conductance regulator (CFTR, OMIM #602421) gene, which is located on chromosome 7q31. The CFTR gene contains 27 exons encoding a glycoprotein (CFTR) of 1480 amino acids, composed of two transmembrane domains (TMD1 and TMD2), two cytosolic nucleotide-binding domains (NBD1 and NBD2), and a phosphorylation-dependent regulatory domain (R) [3]. CFTR dysfunction results in disrupted epithelial homeostasis of water, chloride, sodium, and bicarbonate across various organs and systems [4]. As a result of this, patients with CF present a variable clinical spectrum in which lung damage is the main cause of morbidity and mortality, due to the accumulation of abnormally thick and sticky mucus, leading to recurrent infections from *Sthapylococcus aureus* and *Pseudomonas aeruginosa* bacteria. Additionally, CF patients can present with pancreatic insufficiency (PI), malnutrition, meconium ileus (10–20%), and male infertility due to congenital bilateral absence of the vas deferens (CBAVD) [5]. The life expectancy of CF patients in developed countries is 50 years, while in Mexico it is 21.37 years [6].

In high-income countries, such as the USA, France, Italy, the UK, and Australia, newborn screening (NBS) has been quite successful for CF presymptomatic detection and diagnosis. For example, in populations with Northwestern European ancestry, NBS prognostic values are from 10 to 30% [7]. Despite this, in developing countries, CF diagnosis is traditionally established based on the family’s clinical history and the classic characteristics of the disease, such as the presence of chronic obstructive pulmonary disease, IP, elevated sweat chloride ≥ 60 mmol/L, and molecular confirmation with the presence of 2 PVs in the CFTR gene [8]. In this sense, more than 2000 variants distributed throughout the CFTR gene have been reported, of which 1085 are PV or disease-causing, and 55 variants with variable clinical consequences (http://www.cftr2.org, accessed on 5 September 2024) [9]. The most common PV is p.(Phe508del) (F508del, c.1521_1523del, rs121909001), which is a deletion of the amino acid phenylalanine at position 508 of the protein. The frequency of this variant is similar in populations from the USA, Canada, and Australia since they share an origin with Northwestern European populations [7]. In fact, it is present in 100% of CF patients from the Faroe Islands of Denmark, but in other European countries, such as Italy, its frequency is 67.9%. In American populations, its frequency varies from 25 to 85.8% [7]. The particular genetic admixture of Latino populations has given rise to a wide spectrum of PVs, some of which are exclusive to some countries or even to some families, and Mexico is no exception, with a reported 95 PVs in patients with CF to date [2,6].

Usually, molecular diagnosis of CF has focused on identifying directly the 2 PVs inherited from each parent. However, with advances in next-generation sequencing (NGS) providing complete CFTR sequences, it is now possible to analyze additional variants in the gene, such as variants in the same allele (*in cis*) that can form complex alleles [10]. The study of these complex alleles will allow a deeper understanding of the disease and its broad spectrum of clinical manifestations.

Previous reports have shown that the combination of multiple variants, such as complex alleles, could worsen or mitigate the patient’s clinical condition and potentially influence the structure and function of the CFTR protein, leading to differential responses to CFTR modulator treatment. An example of this was reported in 2020 by Chevalier’s group with the p.[Arg74Trp;Val201Met;Asp1270Asn] (R74W;V201M;D1270N) (Table S1) complex allele. This allele produces a protein with a partial loss of exon 3 with a reduced function. A single (p.Asp1270Asn) variant, when present with another PV in a compound heterozygous state, is not enough to cause CFTR channel dysfunction. However, individuals with the p.[Arg74Trp;Asp1270Asn] combination in the same allele exhibit a minor functional defect, whereas p.[Arg74Trp;Val201Met;Asp1270Asn] (Table S1) leads to mild CF [10]. Similarly, the p.Leu997Phe (L997F) PV could be correlated with CFTR-related disorders (CFTR-RD), but when it is presented as a complex allele with the variant p.(Arg117Leu) (R117L) (Table S1), this combination could produce a mild CF phenotype [11].

On another hand, the effect of *in cis* variants with p.Phe508del has also been reported. The p.[L467F;F508del] complex allele leads to a dysfunction of the CFTR protein with negative effects on the response to lumacaftor. Moreover, patients carrying the p.Phe508del/p.[Phe508del;Phe87Leu;Ile1027Thr] allele show no response to treatment with ivacaftor + lumacaftor; therefore, the triple combination (ivacaftor/tezacaftor/elexacaftor) is recommended [12]. Another variant studied was p.Ala238Val, *in cis* with p.Phe508del, which has been observed to be associated with more severe lung damage and resistance to targeted therapy in CF patients carrying these alleles compared to patients who only carry p.Phe508del [13].

In this study, we investigated the impact of the p.[Ile148Thr;Ile1023_Val1024del] (I148T;3199del6) (Table S1) complex allele, present in nine CF patients. Because these patients are originally from the central region of Mexico and considering the historical background of the migratory flow of the population, we suggest a possible founder effect of this complex allele. For the first time, we reported two homozygous patients carrying this complex allele, and we modeled the encoded protein to better understand the structural dysfunction that it causes. 

## 2. Materials and Methods

We included nine Mexican patients with positive sweat chloride tests (>60 mmol/L) from six unrelated families. DNA extraction was whole venous blood with the QIAmp DNA Blood Maxi kit (Qiagen, Valencia, CA, USA) according to the manufacturer’s protocol. Previously, these patients were sequenced using Multiplicon CFTR Master DX (Agilent, Santa Clara, CA, USA) on the MiSeq system (Illumina, Inc., San Diego, CA, USA), which amplified all exons plus 30 bp of their 5′ and 3′ flanking sequences and UTRs regions. The analysis of CFTR sequencing was done using the MASTR Reporter software v.1.2.1 (Agilent, USA). All variants of the complex allele were validated in the patients through automated Sanger sequencing using the Big Dye Terminator (Applied Biosystems TM, Foster City, CA, USA), and the identification of the carrier status was screened in all their relatives by the same method. This study was conducted in accordance with the Helsinki Declaration and approved by the Ethics and Research Committees of the National Institute for Genomic Medicine (INMEGEN CEI 2015/10). Written informed consent and assent were obtained from all participating families.

The clinical characteristics of the patients were collected from their medical histories and the CF database of our laboratory. The birthplace of families was established through interviews, and family trees were constructed using the Progeny free online pedigree program (https://pedigree.progenygenetics.com (accessed on 10 June 2024)) [14].

The complex allele frequencies were obtained by searches in three different databases (CFTR [CFTR-France (https://cftr.iurc.montp.inserm.fr/cftr/ (accessed on 10 July 2024))] [15], gnomAD [https://gnomad.broadinstitute.org/ (accessed on 10 July 2024)] [16], and CFTR2 [http://www.cftr2.org/ (accessed on 10 July 2024)] [9] and from a dataset in our laboratory comprising 2217 exomes from unrelated adults (Mestizos and Mexican Amerindians) without a history of Mendelian diseases and recruited throughout the Mexican territory [17,18].

We used *in silico* methods to assess the impact of each variant and the complex allele on the CFTR protein. SWISS-MODEL (https://swissmodel.expasy.org/ (accessed on 10 July 2024)) [19] and I-TASSER (https://zhanggroup.org/I-TASSER/ (accessed on 10 July 2024)) [20] were employed to predict the structural effects of the complex allele on the CFTR protein. SIFT-Indel (https://sift.bii.a-star.edu.sg/ (accessed on 10 July 2024)) [21] and Mutation Taster (https://www.mutationtaster.org/ (accessed on 10 July 2024)) [22] were used to assess the functional consequences of p.(Ile1023_Val1024del) and p.(Ile148Thr). DynaMut (https://biosig.lab.uq.edu.au/dynamut/ (accessed on 10 July 2024)) [23] allowed us to analyze changes in protein stability and disruptions in interatomic interactions caused by both individual variants and the complex allele. Protein stability was assessed through ΔΔG estimation, which represents the difference in ΔG between the wild-type and mutant proteins. The CFTR models were uploaded into PyMOL (https://www.pymol.org/ (accessed on 10 July 2024)) [24], where the align command was used to superimpose the mutant structures onto the wild-type structure (PDB ID: 5UAK). The domains of interest [NBD1, NBD2, R] along with residues 148, 1023, and 1024 of CFTR, were highlighted based on curated domain information from Uniprot and Swiss-Prot.

## 3. Results

**Figure 1:**
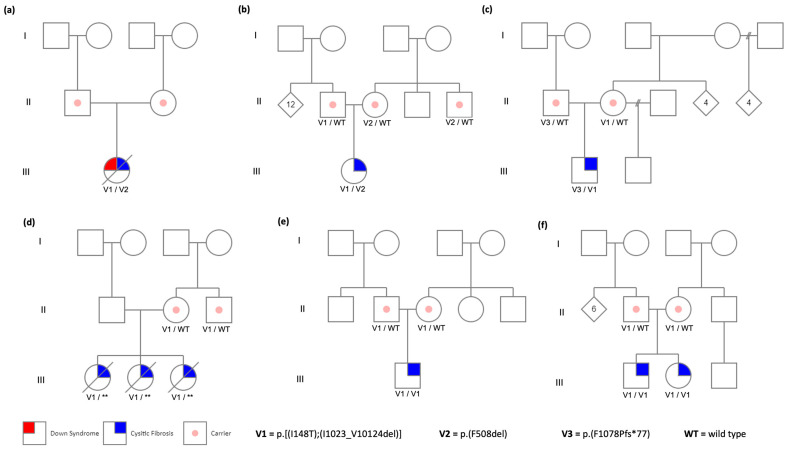
Three-generation pedigree of the six studied families from Central Mexico: (**a**) Family 1, (**b**) Family 2, (**c**) Family 3, (**d**) Family 4, (**e**) Family 5, and (**f**) Family 6. ** depicts that the second affected allele was not detected.

A total of nine CF patients from six unrelated families carrying the complex allele p.[Ile148Thr;Ile1023_Val1024del] were studied. In all families, this complex allele was found *in trans* with the Class I and II PVs [p.(Phe508del) and, p.(Phe1078Profs*77)] (Figure 1a–c). In one family, the second PV could not be identified, possibly due to a deep intronic variant (Figure 1d). Families 5 and 6, declared as nonconsanguineous, carried the complex allele in a homozygous state (Figure 1e,f).

**Figure 2:**
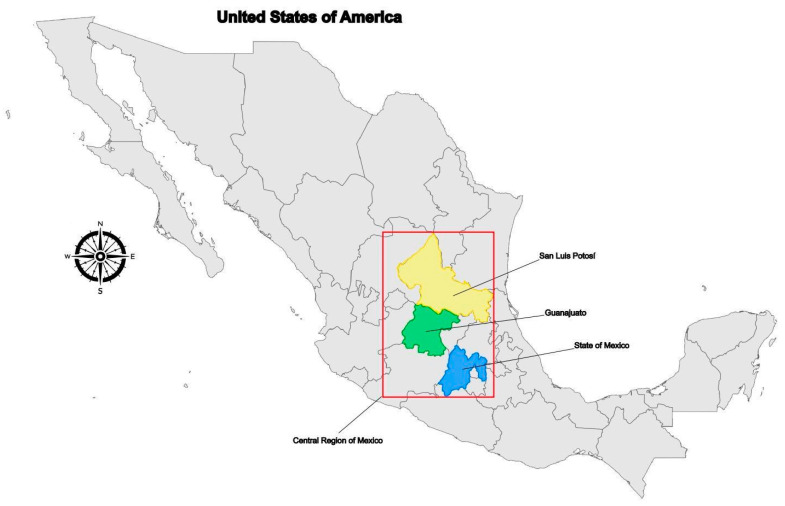
Geographical distribution of the six unrelated families carrying the CFTR complex allele in the Mexican territory. Families are distributed in states from the central region of Mexico depicted by the red box: Guanajuato (green), San Luis Potosí (yellow), and the State of Mexico (blue).

All patients presented severe clinical symptoms characterized by pancreatic insufficiency (PI) and elevated sweat chloride levels (≥85 mEq/L), with diagnosis at birth or less than 4 years of age. Clinical history revealed that all patients’ ancestors were born in Mexico and the Guanajuato State (Central Mexico) (Figure 2).

To further investigate the frequency of variants forming the complex allele, we performed a targeted search in our laboratory dataset containing exome sequences from 2217 individuals. This revealed eight individuals (0.18%) carrying the p.(Ile148Thr) variant, but only one (0.022%), who was also born in Central Mexico (the San Luis Potosi state), carrying the complex allele (Figure 2).

**Figure 3:**
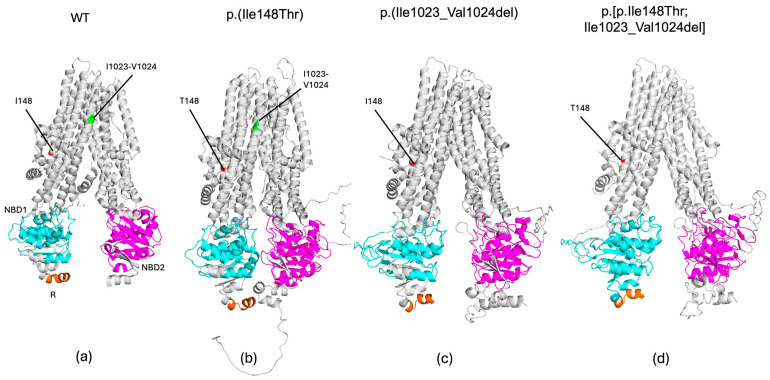
3D structural models of the variants that form the complex allele, alone or in combination. TMD1 and TMD2 domains are depicted in gray, NBD1 and NBD2 domains are in blue and pink, respectively, and the R domain is indicated in orange. (**a**) Wild-type CFTR structure (5uak.pdb) with the position of the variants analyzed: p.(Ile148Thr) (I148T) in red and p.(Ile 1023_Val1024del) (3199del6) in green. Modeling of the resulting protein carrying the (**b**) p.(Ile148Thr) variant, (**c**) p.(Ile 1023_Val1024del), and (**d**) p.[p.Ile148Thr;Ile1023_Val1024del] complex allele.

To better our understanding of the structural impact of each variant and the complex allele on the CFTR protein, we performed an *in silico* analysis (Figure 3). Modeling of the p.(Ile148Thr) variant displayed a destabilized protein, with a predicted stability change (ΔΔG Stability wt to mut = −2.05 kcal/mol). MutationTaster also classified it as disease-causing (probability: 99.9%). Protein modeling of this variant revealed a significant structural disruption in CFTR (Figure 3b). The Ile148 is located at the junction between the second and third helixes of TMD1 near the interface between the membrane and the cytoplasm. The substitution of this isoleucine with threonine (148Thr) alters the interaction of helixes 1 and 2, and the cytoplasmic loops 1 and 2 in TMD1 in the protein. Additionally, the change to 148Thr induces changes in the cytoplasmic side of the protein compatible with a narrowing of the cytoplasmic side of channel pore (Figure 3b).

The SIFT-INDEL tool predicted a confidence score of 0.858 for the p.(Ile1023_Val1024del) variant, indicating a damaging effect. Additionally, MutationTaster classified this variant as disease-causing with a 71% probability. Furthermore, protein modeling revealed that this variant disrupts the secondary structure of CFTR. Specifically, it affects helix 10 of TMD2, which is crucial for its stabilization, configuration, opening and closing of the channel pore. This deletion also disrupts the helical structure of the helix 10, potentially destabilizing the entire transmembrane domain. The structural analysis also predicted a misconfiguration of the protein in the NBD1 and NBD2 domains (Figure 3c).

Finally, when both variants were modeled together, as a complex allele, the resulting protein conformation exhibited more pronounced changes, including a narrowing cytoplasmic face of the channel pore, rearrangement of the NBD1, and NBD2 domains, as well as the loss of part of the helix 10. In consequence, the combination of p.(Ile148Thr) and p.(Ile1023_Val1024del) variants might alter the ion flow (Figure 3d).

## 4. Discussion

Traditionally, CF diagnosis has relied on identifying two PVs *in trans*, one inherited from each parent. Advances in sequencing technologies have enabled the detection of rare CFTR variants, including complex alleles. First described in 1991, complex CFTR alleles are characterized by the presence of two or more variants *in cis*, creating a significant challenge for CF diagnosis [4,10]. Such alleles may have significant implications for CFTR function and the clinical manifestation of the disease [25].

Previously, we screened 297 CF patients across Mexico [6]. Notably, the complex allele p.[Ile148Thr;Ile1023_Val1024del] was identified in nine patients from six different families, all of them born in the central region of Mexico. Here, we report for the first time two CF patients homozygous for that complex allele. All nine patients exhibited severe clinical symptoms.

Notably, p.(Ile1023_Val1024del) has been reported as a CF-causing variant, while p.(Ile148Thr) has been classified as a neutral, common CFTR variant that does not affect the pathogenicity of additional variants in complex alleles [26]. The classification of these variants is in line with the CFTR (http://www.cftr2.org/ (accessed on 10 July 2024) and https://cftr.iurc.montp.inserm.fr (accessed on 10 July 2024)) databases [9,15].

In this study, we documented the structural impact of a complex allele and each independent variant on the CFTR protein using an *in silico* analysis. The protein conformation derived from the complex allele showed more severe changes compared with the conformation associated with each independent variant. The p.(Ile1023_Val1024del) variant, which involves a deletion of the ATAGTG sequence in CFTR exon 19, results in the removal of isoleucine and valine at residues 1023 and 1024 in the TMD2 domain [27,28]. In addition, protein modeling revealed that this variant disrupts the secondary structure of CFTR, particularly affecting helix 10 of TMD2, which is essential for its stabilization. This analysis also showed misfolding affecting the NBD2 domain and the transmembrane domain connecting TMD2 and NBD2, suggesting that channel ion flow may be reduced.

Otherwise, modeling of the p.(Ile148Thr) variant indicates that it destabilizes the CFTR protein (ΔΔG Stability = −2.05 kcal/mol). Ile148 is located at the junction of the first and second helixes of TMD1 near the interface between the membrane and the cytoplasm. The substitution of Ile by Thr may significantly impact the protein’s structure, stability, and interactions due to the significant differences in hydrophobicity and polarity between these two amino acids. In fact, we found that protein modeling with this amino acid substitution had a significant effect on the configuration of the TMD1 and NBD1 domains of CFTR. Furthermore, the MutationTaster tool classified this variant as disease-causing with a 99.9% probability. Supporting this, a previous functional study showed that nasal epithelial cells (NEC) from individuals carrying p.(Ile148Thr) exhibit a CFTR gating activity of 86–87.4% and induce in patients a variable spectrum of clinical characteristics with null or mild effects, displaying mild channel impairment [26]. Perhaps this dysfunction is not enough to cause CF manifestations, although it has been reported that in some patients, this variant *in trans* with a PV may result in CF-like symptoms [29] or result in a diagnosis of a CFTR-related disorder [27].

Finally, our modeling suggests that the p.(Ile148Thr) and p.[Ile1023_Val1024del] variants *in cis* synergistically affect protein conformation, narrowing the channel and thus altering the ion flow even more, as previously documented in individuals carrying the complex allele along with other Class I and II variants, who displayed a CFTR gating activity on NEC of 7.3% [26,29].

A growing body of evidence has demonstrated that patients carrying complex alleles could have altered CFTR biogenesis, inducing a variable spectrum of clinical characteristics which makes diagnosis more complicated. Furthermore, the presence of these alleles could induce a different response to the target drugs, such as elexacaftor/ivacaftor/tezacaftor, highlighting the importance of performing the CFTR-NGS in all CF patients for accurate diagnosis and for personalizing therapeutic strategies to improve patient outcomes [4,30].

To estimate the frequency of the complex allele in Mexico, we searched our laboratory’s exome-sequencing dataset for carriers of the complex allele, as well as the individual variants p.(Ile148Thr) and p.(Ile1023_Val1024del). We identified no carriers of the p.(Ile1023_Val1024del), eight carriers of the p.(Ile148Thr), and the complex allele in only one individual, also of central Mexican origin, suggesting a possible enrichment of this variant in the central region of Mexico. The CFTR-France database shows that 23.53% of subjects carrying p.(IleI148Thr) are also carriers of the 3199del6 variant as a complex allele [15]. Moreover, it has been reported that this allele is present in 2.2% of CF patients from Quebec City, Canada [28]. In our patients, the p.[Ile148Thr;Ile1023_Val1024del] allele was present at a frequency of 0.18%. Notably, this complex allele has not been reported in other populations and is absent from the CFTR2 database, highlighting the importance of these results. Historical records indicate that French-Canadian immigrants settled in Mexico in the late 19th century, mainly in the cities of Central Mexico, driven by the growth of industries such as electrical infrastructure, mining, and railways [31,32]. This context, coupled with the geographic origin of the complex allele carriers, allows us to suggest that this allele may be of French origin and was introduced to America through the French-Canadian population, who much later brought it into the population of Central Mexico, supporting our hypothesis of a possible founder effect; however, studies of genetic markers are necessary to confirm this hypothesis.

## 5. Conclusions

Our study provides evidence that the p.[Ile148Thr;Ile1023_Val1024del] complex allele is enriched in the central region of Mexico, suggesting a possible founder effect in Mexican patients with CF. It also shows that the p.(Ile148Thr) variant has a functional effect, highlighting the importance of monitoring the clinical outcomes of patients carrying this variant to fully understand its implications. Additionally, our findings support the integration of NGS into CF diagnostics to ensure the identification of all CF alleles, including complex alleles such as p.[Ile148Thr;Ile1023_Val1024del], which are associated with severe clinical manifestations, including early mortality. NGS diagnosis is crucial for developing personalized treatment strategies and improving patient quality of life.

## Data Availability

Data are contained within the article and Supplementary Materials.

## Supplementary Materials

The following supporting information can be downloaded at: https://www.mdpi.com/article/10.3390/life14111445/s1, Table S1: Nomenclature of the CFTR variants mentioned in the manuscript.
