# Supplementary material for: Effect of the Complex Allele p.[Ile148Thr;Ile1023_Val1024del] in Cystic Fibrosis and Tracing of a Founder Effect in Mexican Families
Source: Life (Basel). 2024 Nov 7;14(11):1445. doi: 10.3390/life14111445 (PMC11596012; doi:10.3390/life14111445)
Supplement: Supplementary file 1 [file life-14-01445-s001.zip › life-3246450-supplementary.pdf]

Table S1. Nomenclature of the variants mentioned in the manuscript.

	Legacy Name	cDNA	Protein
1	R74W	c.220C>T	p.(Arg74Trp)
2	F87L	c.259T>C	p.(Phe87Leu)
3	R117L	c.350G>T	p.(Arg117Leu)
4	I148T	c.443T>C	p.(Ile148Thr)
5	V201M	c.601G>A	p.(Val201Met)
6	1531C/T (L467F)	c.1399C>T	p.(Leu467Phe)
7	L997F	c.2991G>C	p.(Leu997Phe)
8	3199del6	c.3067_3072del	p.(Ile1023_Val1024del)
9	ΔF508	c.1521_1523del	p.(Phe508del)
10	c.3231_3232delGT	c.3231_3232del	p.(Phe1078Profs*77)
11	D1270	c.3808G>A	p.(Asp1270Asn)
